# Heterologous prime-boost immunisation with mRNA- and AdC68-based 2019-nCoV variant vaccines induces broad-spectrum immune responses in mice

**DOI:** 10.3389/fimmu.2023.1142394

**Published:** 2023-03-15

**Authors:** Xingxing Li, Jingjing Liu, Wenjuan Li, Qinhua Peng, Miao Li, Zhifang Ying, Zelun Zhang, Xinyu Liu, Xiaohong Wu, Danhua Zhao, Lihong Yang, Shouchun Cao, Yanqiu Huang, Leitai Shi, Hongshan Xu, Yunpeng Wang, Guangzhi Yue, Yue Suo, Jianhui Nie, Weijin Huang, Jia Li, Yuhua Li

**Affiliations:** ^1^ Department of Arboviral Vaccine, National Institutes for Food and Drug Control, Beijing, China; ^2^ State Key Laboratory of Biotherapy and Cancer Center, West China Hospital, Sichuan University, and Collaborative Innovation Center for Biotherapy, Chengdu, China; ^3^ Department of Respiratory Virus Vaccine, National Institutes for Food and Drug Control, Beijing, China; ^4^ Department of HIV/AIDS and Sex-transmitted Virus Vaccines, National Institutes for Food and Drug Control, Beijing, China

**Keywords:** heterologous prime-boost, ChAdTS-S, ARCoV, intramuscular, intranasal, SARS-COV-2 variants, COVID-19

## Abstract

The ongoing evolution of severe acute respiratory syndrome coronavirus 2 (SARS-CoV-2 or 2019-nCoV) variants has been associated with the transmission and pathogenicity of COVID-19. Therefore, exploring the optimal immunisation strategy to improve the broad-spectrum cross-protection ability of COVID-19 vaccines is of great significance. Herein, we assessed different heterologous prime-boost strategies with chimpanzee adenovirus vector-based COVID-19 vaccines plus Wuhan-Hu-1 (WH-1) strain (AdW) and Beta variant (AdB) and mRNA-based COVID-19 vaccines plus WH-1 strain (ARW) and Omicron (B.1.1.529) variant (ARO) in 6-week-old female BALB/c mice. AdW and AdB were administered intramuscularly or intranasally, while ARW and ARO were administered intramuscularly. Intranasal or intramuscular vaccination with AdB followed by ARO booster exhibited the highest levels of cross-reactive IgG, pseudovirus-neutralising antibody (PNAb) responses, and angiotensin-converting enzyme-2 (ACE2)-binding inhibition rates against different 2019-nCoV variants among all vaccination groups. Moreover, intranasal AdB vaccination followed by ARO induced higher levels of IgA and neutralising antibody responses against live 2019-nCoV than intramuscular AdB vaccination followed by ARO. A single dose of AdB administered intranasally or intramuscularly induced broader cross-NAb responses than AdW. Th1-biased cellular immune response was induced in all vaccination groups. Intramuscular vaccination-only groups exhibited higher levels of Th1 cytokines than intranasal vaccination-only and intranasal vaccination-containing groups. However, no obvious differences were found in the levels of Th2 cytokines between the control and all vaccination groups. Our findings provide a basis for exploring vaccination strategies against different 2019-nCoV variants to achieve high broad-spectrum immune efficacy.

## 1 Introduction

Severe acute respiratory syndrome coronavirus 2 (SARS-CoV-2 or 2019-nCoV) is a highly mutable, enveloped, single-stranded RNA coronavirus of the betacoronavirus genus (1). According to the World Health Organization, the five variants of concern (VOCs), such as Omicron (e.g., BQ.1, BA.5, BA.2.75, BA.2, B.1.1.529 and BA.1), Gamma (P.1), Delta (B.1.617.2), Beta (B.1.351) and Alpha (B.1.1.7), have all caused COVID-19 pandemic waves at varying magnitudes (2–4). At present, the most prevalent global 2019-nCoV variant is the Omicron variant, with > 30 mutations in the spike protein and 15 point mutations in the spike receptor-binding domain (RBD) region compared to the Wuhan-Hu-1 (WH-1) strain (5). Omicron is more transmissible and can evade immunity more efficiently than the other VOCs, thereby increasing the risk of reinfection (6). Two doses of Pfizer/BNT162b2 mRNA vaccine elicited a 22-fold lower serum neutralising activity against Omicron (B.1.1.529) than against the D614G variants (7). Two doses of BNT162b2 or ChAdOx1 nCoV-19 had limited protection against symptomatic B.1.1.529 infection, with vaccine efficacy decreasing to <10% at 20 weeks or beyond (8). Thus, it is of utmost urgency to develop COVID-19 vaccines that are more effective against emerging variants.

Novel 2019-nCoV variant vaccines induce immune responses against different 2019-nCoV variants in mice (9–12). Although Omicron-specific vaccines induce high neutralising activity against Omicron, they are not effective against other variants (9, 10). COVID-19 vaccines expressing spike proteins from only Beta or Delta variants showed low immunogenicity, but induced relatively broad-spectrum neutralising antibodies against multiple variants (9, 10, 12). Moreover, the nucleocapsid (N) protein is highly conserved and immunogenic among 2019-nCoV variants and other coronaviruses. The plant-produced RBD and cocktail-based vaccine (RBD co-expressed with N protein) is highly effective against 2019-nCoV variants, such as Omicron and Delta variants, in mice (13). Higher systemic immune response and protective efficacy have been induced using a heterologous, rather than a homologous, prime-boost strategy (14, 15). We previously found that intranasal (in) or intramuscular (im) priming with ChAdTS-S and im boosting with ARCoV induced greater cellular and humoral immune responses than homologous vaccination with either vaccine (16). This vaccination strategy could potentially prevent immune escape of the current 2019-nCoV variants, especially Omicron strains. Thus, it is necessary to explore a new heterologous prime-boost strategy with different novel 2019-nCoV variant vaccines for improving broad-spectrum neutralising potency against the current and emerging variants.

Mucosal immune response (MIR) plays indispensable roles in fighting and preventing respiratory viral infections, and the high level of secreted IgA (SIgA) during MIR can significantly improve the neutralisation efficacy (17, 18). Polymeric secretory IgA (psIgA) antibodies against influenza A viruses of multiple hemagglutinin (HA) subtypes do not exhibit neutralising properties, but have broad cross-binding and protective capacities (19). Therefore, enhancing the vaccine-induced MIR might be crucial for preventing infection caused by different 2019-nCoV variants.

Herein, we explored the heterologous prime-boost strategies using chimpanzee adenovirus serotype 68 vector (AdC68)- and mRNA-based COVID-19 vaccines with different vaccination routes against different 2019-nCoV variants [prototype, Beta, and Omicron (B.1.1.529) strains]. Our study provides reference data for improving the spectrum and immunogenicity of multiple 2019-nCoV variant vaccines.

## 2 Materials and methods

### 2.1 Animals and vaccines

**Figure 1:**
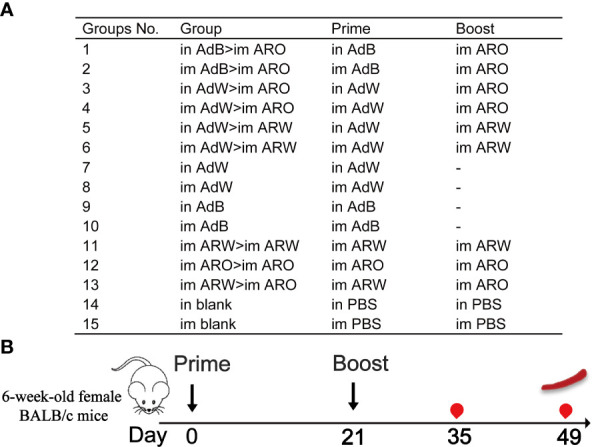
Overall scheme of the immunisation strategies and experimental timeline using female BALB/c mice. **(A)** Mice in 15 groups were immunised with four COVID-19 vaccines using different protocols. Dashes indicate no booster vaccination. **(B)** Immunisation and immunological characterisation scheme. 
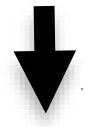
, vaccination; 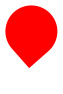, bleeding; 
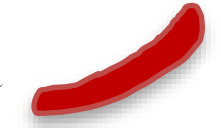
, spleen lymphocyte isolation. Im, intramuscular vaccination; in, intranasal vaccination.

Six-week-old female BALB/c mice (pathogen-free) were obtained and maintained in the National Institutes for Food and Drug Control (NIFDC). We evaluated the chimpanzee adenovirus vector 2019-nCoV vaccine ChAdTS-S (5 × 10^10^ vp/0.5 mL, Walvax, Yunnan, China) was evaluated, which encoded the WH-1 strain spike protein and designated as AdW; chimpanzee adenovirus vector vaccine ChAdTS-SV2 (5 × 10^10^ vp/0.5 mL, Walvax), which encoded the Beta strain spike protein and designated as AdB; mRNA vaccine ARCoV (15 µg/0.5 mL, Abogen, Suzhou, China), which encoded the WH-1 strain spike RBD and designated as ARW; mRNA vaccine ARCoV-Omicron (15 µg/0.5 mL, Abogen) and designated as ARO, which encoded the Omicron variant B.1.1.529 with RBD mutations (20). The mice were randomly assigned to groups 1–6, 7–10, 11–13 and 14–15 (n = 5 per group) that received heterologous prime-boost, single doses, homologous prime-boost vaccinations, and phosphate-buffered saline (PBS, blank control), respectively (**Figures 1A, B**). Initial vaccination was administered on day 0, and booster vaccination was administered on day 21. The inoculation was a fractional dose (one-fifth of the human dose), i.e., 1 × 10^10^ vp per mouse for AdW and AdB and 3 µg per mouse for ARW and ARO. Blood specimen was withdrawn from each mouse 35 and 49 days after the initial vaccination. This research was approved by the Institutional Animal Care and Use Committee of NIFDC, and was performed in compliance with the Committee guidelines.

### 2.2 Enzyme-linked immunosorbent assay

2019-nCoV spike protein-specific IgA and IgG titres were detected with ELISA. Briefly, 0.2 μg 2019-nCoV spike protein (Sino Biological, Beijing, China), and B.1.1.529 spike protein (Sino Biological) were coated overnight onto the Costar ELISA plates (Corning, NY, USA), respectively. The 2019-nCoV spike protein was fused with a polyhistidine tag at the C-terminus, while the B.1.1.529 spike protein was fused with the bacteriophage T4 fibritin and a polyhistidine tag at the C-terminus. After blocking with 0.05% Tween 20-containing PBS and 1% bovine serum albumin at 37°C for 1 h, the plates were rinsed 6 times with 0.05% Tween 20-containing PBS, the diluted sera were added to the wells by 4-fold serial dilutions. After washing 6 times with 0.05% Tween 20-containing PBS, the plates were exposed to horseradish peroxidase-conjugated goat anti-mouse IgA (1:10,000; Abcam, UK) or goat anti-mouse IgG (1:10,000; ZSGB-BIO, China) at 37°C for 1 h. TMB (3,3’,5,5’-tetramethylbenzidine; Beyotime, China) was employed as a substrate to determine the antibody responses by measuring the absorbance at 450 and 630 nm. The end point titres were defined as the highest reciprocal serum dilution, which was 2.1-fold higher compared to the negative control.

### 2.3 Recombinant vesicular stomatitis virus-based pseudovirus neutralisation assay

Recombinant VSV-based pseudotyped 2019-nCoV was obtained from the Division of HIV/AIDS and Sex-Transmitted Virus Vaccines, NIFDC, including Delta (B.1.617.2) variant, Omicron (B.1.1.529) variant, Omicron (BA.4/5) variant and WH-1 strain. The assays were conducted according to a previous method (21). After inactivation at 56°C for 30 min, a 3-fold serial dilution of mouse serum was mixed with 650 median tissue culture infectious dose (TCID_50_) of pseudoviruses, followed by incubation at 37°C for 60 min. Vero cells (2 × 10^5^) were added, followed by incubation at 37°C and 5% CO_2_ for 24 h. NAbs were detected by luciferase expression to determine the amount of pseudoviruses entering the target cells. A luciferase assay system (PerkinElmer, MA, USA) was used to detect the luciferase activity. We included a virus control containing both virus and cells and a negative control containing only cells. The half-maximal effective concentration was determined for each tested sample. For neutralising titre <30, the value was recorded as 30 for plotting.

### 2.4 Live SARS-CoV-2 neutralisation assay

The neutralising capacity of mouse sera was evaluated using a microneutralisation (MN) assay. Two-fold serial dilutions of heat-inactivated sera were exposed to 100 median cell culture infectious dose (CCID_50_) of 2019-nCoV CAS-B001/2020 strain at 37°C for 2 h. Vero E6 cells (1.8 × 10^5^) were added, followed by incubation at 37°C for 72 h. The MN antibody titres were calculated using the Spearman–Karber method to assess the serum dilution needed for 50% inhibitory action of the cytopathic activity, and the MN antibody titre of ≥ 4 was regarded as positive. Each dilution was carried out in duplicate. The virus culture of 2019-nCoV and the MN assays was conducted in a biosafety level-3 facility at NIFDC.

### 2.5 ACE2-binding inhibition (neutralisation) ELISA

The V-PLEX COVID-19 ACE2 neutralisation kit (Panel 18 (ACE2) kit, K15570U; Panel 27 (ACE2) kit, K15609U-2; Meso Scale Discovery, Rockville, MD, USA) was employed to quantitatively analyze of antibody titres that blocked the binding of ACE2 to its cognate ligands (spike protein from BA.5, BA.4, BA.3, BA.2+L452R, BA.2+L452M, BA.2.12.1, BA.2, P.2, P.1, B.1.617.3, B.1.617.2, B.1.617.1, B.1.617, B.1.526.1, B.1.351, B.1.1.7 and WH-1 strains). The 96-well plate was pre-coated with the specific antigen on spots, and the bound antibodies in each sample (1:100 dilution) were analyzed by human ACE2 protein conjugated with the Meso Scale Discovery (MSD) SULFO-TAG using the MSD instrument. The ACE2-binding inhibition rate was calculated as: 1 - (average electrochemiluminescence signal value of sample - average electrochemiluminescence signal value of blank) × 100%.

### 2.6 IFN-γ ELISpot assay

Mice were euthanised and immersed in 75% ethanol. The spleen was collected and transferred into a 40-μm cell strainer. Then, 4–5 mL mouse lymphocyte separation medium (Dakewe, China) was added. After grinding with a 2-mL syringe piston, the spleen cell suspension was placed in a 15-mL centrifuge tube and added with 1 mL RPMI-1640 medium (Hyclone, Logan, UT, USA). After centrifugation (800 × *g*, 30 min), the mixture was classified into 4 layers from bottom to top: the cell fragment and erythrocyte layer, lymphocyte layer, fluid separation layer, and RPMI-1640 medium layer. The lymphocytes were transferred to a fresh tube, and then added with 10 mL RPMI-1640 medium. After centrifugation (250 × *g*, 10 min), lymphocytes were harvested and suspended in serum-free medium (Dakewe). A mouse IFN-γ ELISpot plus kit (Mabtech, Sweden) was used to detect IFN-γ-positive cells. Briefly, the plates were rinsed 4 times with 1× PBS (200 µL) and blocked with 10% FBS-containing RPMI-1640 medium at 24°C for 2 h. The freshly isolated lymphocytes (2.5 × 10^5^) were incubated with a peptide pool (1 µg/mL per peptide, Genscript, Nanjing, China) obtained from a peptide scan (15-mers with 11-residue overlaps) of the whole spike glycoprotein of B.1.1.529, B.1.617.2 and 2019-nCoV at 37°C and 5% CO_2_ and for 24 h. After incubation with anti-mouse IFN-γ antibody for 2 h, the plates were incubated again with streptavidin-horseradish peroxidase (1:1,000 dilution, Dakewe) for 1 h. TMB solution (100 μL) was added into each well and developed for 5 min until the appearance of different spots. The ImmunoSpot^®^ S6 Universal instrument (Cellular Technology Limited, USA) was used to observe and count the spots.

### 2.7 Intracellular cytokine staining

Splenic lymphocytes were isolated and stimulated with 2 μg/mL of the spike protein peptide pool and brefeldin A (1:1,000 dilution, Biolegend, USA) at 37°C and 5% CO_2_ for 6 h. After stimulation, the splenocytes were rinsed and stained with fixable viability stain and 780 the following antibodies: FITC rat anti-mouse CD8a antibody, BV510 rat anti-mouse CD4 antibody and BV421 hamster anti-mouse CD3e antibody (BD Biosciences, USA). The cells were rinsed twice with 1× PBS, fixed and permeabilised with Cytofix/Cytoperm (BD Biosciences). After rinsing with Perm/Wash buffer (BD Biosciences), the cells were stained with BB700 rat anti-mouse tumour necrosis factor (TNF), APC rat anti-mouse IL-10, PE-Cy7 rat anti-mouse IL-4, BV605 rat anti-mouse interleukin (IL)-2 and PE-conjugated rat anti-mouse IFN-γ (BD Biosciences). The cells were successively rinsed with Perm/Wash buffer, resuspended in 1× PBS, and determined using a FACS Lyric flow cytometric analyser (BD Biosciences). For each sample, 200,000 events were recorded, and data analysis was performed with FlowJo software (TreeStar, USA). CD4^+^ and CD8^+^ T cells were obtained by gating single cells (FSC-A versus FSC-H), lymphocytes (FSC-A versus SSC-A), and live CD3^+^ T cells (CD3^+^ versus LD780^−^). All results are expressed as the percentage of cytokine+ cells in CD4^+^ or CD8^+^ T cells.

### 2.8 MSD profiling of Th1/Th2 cytokines

The supernatant was harvested from ELISpot plates, and the levels of IL-2, IL-4, IL-10 and TNF-α were measured using a V-PLEX Proinflammatory Panel 1 (mouse) Kit. Meanwhile, the levels of cytokines were detected using a MESO QuickPlex SQ 120. A standard curve was used to calculate the concentration of each cytokine.

### 2.9 Statistical analysis

GraphPad Prism v9 software was employed for all plotting and statistical tests. Data are shown as the geometric mean ± geometric standard deviation (except for ACE2-binding inhibition rates that are shown as mean ± standard deviation). Differences among multiple groups were compared by one-way ANOVA. *P<0.05; **P<0.01; ***P<0.001; ****P<0.0001; ns, not significant.

## 3 Results

### 3.1 Intranasal or intramuscular administration of AdB followed by ARO immunisation induces strong humoral immune response against multiple 2019-nCoV variants in mice

**Figure 2:**
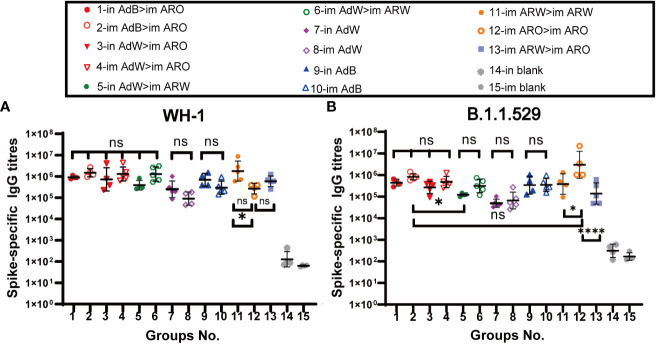
IgG responses induced by AdW, AdB, ARW, and ARO vaccines that were administered using various protocols. All titres were measured on day 35 after primary immunisation. Serum IgG titres against **(A)** WH-1 and **(B)** B.1.1.529 spike protein (n = 4−5 per group; each spot represents one sample). Bars represent the geometric mean ± geometric SD; *P < 0.05; ***P < 0.001; ns, P > 0.05.

The heterologous prime-boost designs are displayed in **Figures 1A, B**. To assess the humoral immune response, the spike-specific IgG titres in serum on day 35 after primary immunisation were detected by ELISA (**Figure 2**). The six heterologous prime-boost immunisation groups (in AdB > im ARO, im AdB > im ARO, in AdW > im ARO, im AdW > im ARO, in AdW > im ARW, and im AdW > im ARW) developed similar high spike-specific IgG antibody titres against the WH-1 strain (**Figure 2A**). However, no obvious differences were found among the six groups. The im AdB > im ARO, im AdW > im ARO, im AdW > im ARW and im ARW > im ARW groups developed the highest IgG geometric mean titres (GMTs) of 1,480,872, 1,299,190, 1,284,719 and 1,771,304, respectively. The IgG GMTs for single-dose vaccinated groups (in AdW, im AdW, in AdB and im AdB) were 248,649, 90,299, 689,171, and 295,854, respectively. AdB administered either intranasally or intramuscularly induced higher IgG GMTs than AdW. im ARW > im ARW had higher IgG titres than im ARO > im ARO and im ARW > im ARO, showing 5.6- and 2.0-fold increases, respectively.

Spike-specific IgG titres against the B.1.1.529 spike protein showed a downward trend compared with those against the WH-1 spike protein (**Figure 2B**), with the six heterologous prime-boost immunisation groups having IgG GMT values of 440,438, 829,265, 266,256, 475,462, 121,796 and 313,054, respectively, indicating a 1.1-, 0.8-, 1.7-, 1.7-, 2.2- and 3.1-fold decrease, respectively. The IgG GMTs for single-dose vaccinated groups against the B.1.1.529 spike protein (in AdW, im AdW, in AdB and im AdB) were 49,082, 66,044, 339,534 and 349,768, showing a 4.1-, 0.4-, and 1.0-fold decrease and a 0.2-fold increase, respectively, compared to those against the WH-1 spike protein. The highest IgG antibody titres were generated by im ARO > im ARO against the B.1.1.529 spike protein among all tested groups, with an IgG GMT of 2,982,389. The IgG GMT in the im ARO > im ARO group was remarkably increased compared to that in the im ARW > im ARW (P = 0.0287) and im ARW > im ARO (P<0.0001) groups, showing a 6.8- and 20.5-fold increase, respectively.

**Figure 3:**
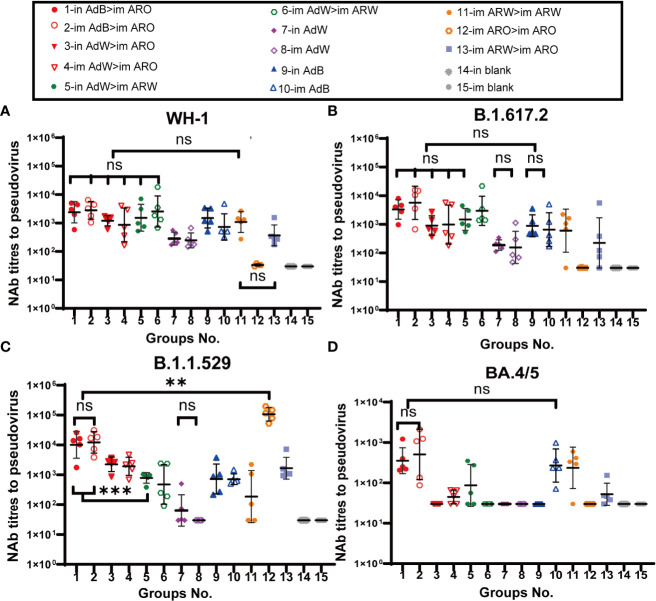
Pseudovirus-neutralising antibody (PNAb) titres were measured 35 days after primary immunisation. Serum NAb titres against **(A)** WH-1, **(B)** B.1.617.2, **(C)** B.1.1.529, and **(D)** BA.4/5 are expressed as 50% inhibitory dilutions (n = 4−5 per group; one spot represents one sample). Bars represent the geometric mean ± geometric SD; **P < 0.01; ***P < 0.001; ns, P > 0.05.

**Figure 4:**
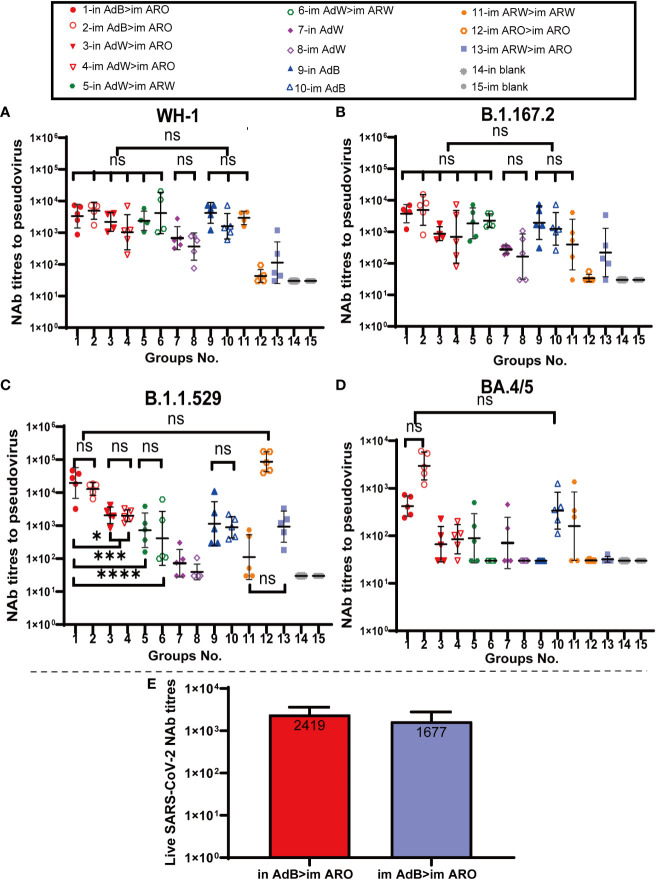
NAb titres were measured 49 days after primary immunisation. Serum PNAb titres against **(A)** WH-1, **(B)** B.1.617.2, **(C)** B.1.1.529, and **(D)** BA.4/5 are expressed as 50% inhibitory dilutions (n = 4−5 per group; one spot represents one sample). **(E)** SARS-CoV-2 NAb titration (n = 3 per group). Bars represent the geometric mean ± geometric SD; *P < 0.05; ***P < 0.001; ****P < 0.0001; ns, P > 0.05.

Serum neutralizing antibody titers against BA.4/5 variant, B.1.1.529 variant, B.1.617.2 variant, and WH-1 strain were evaluated with VSV-based pseudovirus assays on day 35 (**Figures 3A−D**) and 49 (**Figures 4A−D**) after primary immunisation. The six heterologous prime-boost immunisation groups produced relatively high NAb GMTs in response to the WH-1 strain on day 35 (**Figure 3A**), which were not significantly different. The in AdB > im ARO, im AdB > im ARO and im AdW > im ARW groups had the highest NAb titres against the WH-1 pseudovirus among all tested groups, with NAb GMTs of 2,387, 2,806 and 2,536, respectively. Higher NAb levels were induced by in AdB and im AdB compared to in AdW and im AdW, with NAb GMTs of 1,493, 725, 283 and 246, respectively. The NAb GMTs for im ARW > im ARW and im ARW > im ARO were 1,084 and 367, respectively. The im ARO > im ARO group had very low NAb levels against the WH-1 strain when compared to the im and in blank groups.

The neutralizing antibody titres against B.1.617.2 variant were comparable with those against WH-1 strain (**Figure 3B**) in all tested groups. The in AdB > im ARO, im AdB > im ARO, and im AdW > im ARW groups had the highest NAb titres against the B.1.617.2 pseudovirus among all tested groups, with NAb GMTs of 3,308, 5,653 and 2,966, respectively. The NAb GMTs for the in AdW, im AdW, in AdB, im AdB, im ARW > im ARW and im ARW > im ARO groups were 188, 156, 884, 650, 599, and 224 respectively. Of note, im ARO > im ARO induced few NAbs, similar to the im and in blank groups.

In response to the B.1.1.529 pseudovirus on day 35 (**Figure 3C**), AdB followed by ARO (in AdB > im ARO and im AdB > im ARO) induced high NAb titres that were comparable with those induced by im ARO > im ARO, showing NAb GMTs of 10,065, 12,152 and 106,635, respectively. The remaining four heterologous and two homologous prime-boost groups exhibited relatively low NAb titres, with GMTs of 2,237 in in AdW > im ARO, 1,915 in im AdW > im ARO, 785 in in AdW > im ARW, 473 in im AdW > im ARW, 186 in im ARW > im ARW, and 1,661 in im ARW > im ARO. Higher NAb levels were induced with in AdB and im AdB than with in AdW and im AdW, showing NAb GMTs of 723, 712, 64 and 30, respectively.

NAb titres against the BA.4/5 pseudovirus were lower than those against the WH-1 stain (**Figure 3D**). The in AdB > im ARO, im AdB > im ARO, im ARW > im ARW and im ARO > im ARO groups had the highest NAb responses, with NAb GMTs of 354, 504, 268 and 235, respectively, which were 5.8-, 4.6-, 1.7- and 3.6-fold lower than those against WH-1. All remaining vaccination groups induced NAb GMTs comparable with those of the im and in blank groups.

Serum neutralizing antibody titers against the BA.4/5, B.1.1.529, B.1.617.2 and WH-1 pseudoviruses on day 49 (**Figures 4A−D**) after primary immunisation were comparable with those detected on day 35. AdB, followed by ARO groups, induced the broadest spectrum and highest NAb responses in all tested groups against the WH-1 strain, BA.4/5, B.1.1.529 and B.1.617.2 variants, with GMTs of 2,747, 421, 19,557 and 3,763 in in AdB > im ARO and 3,828, 2,951, 12,915 and 4,924 in im AdB > im ARO, respectively. Both im and in AdB induced higher broad-spectrum NAb responses against the four pseudoviruses than in AdW and im AdW. Moreover, im ARO > im ARO induced high NAb responses against its own B.1.1.529 pseudovirus but almost no NAbs against the other three pseudoviruses.

To further assess NAbs for live SARS-CoV-2, NAb titres in serum were detected using a virus-specific MN assay on day 49 (**Figure 4E**) after primary immunisation. The NAb GMTs of the in AdB > im ARO and im AdB > im ARO groups were 2,419 and 1,677, respectively.

Taken together, in AdB > im ARO and im AdB > im ARO induced systemic immune responses with the broadest spectrum, as indicated by the highest IgG and NAb GMTs against the WH-1 strain, BA.4/5, B.1.1.529 and B.1.617.2 variants.

### 3.2 Intranasal administration of AdB followed by ARO immunisation induces high mucosal immune response against WH-1 and B.1.1.529 strains in mice

**Figure 5:**
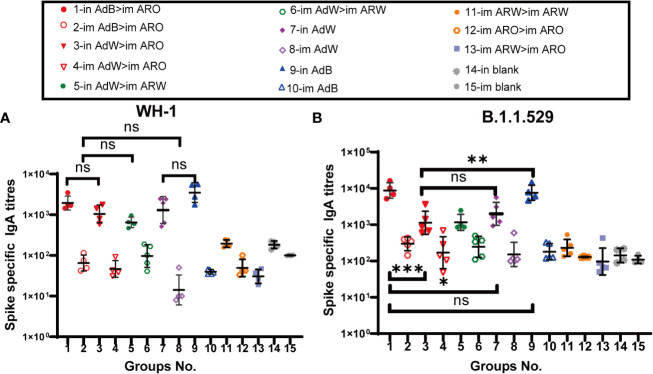
IgA responses induced by AdW, AdB, ARW, and ARO vaccines were administered using various protocols. All titres were measured on day 35 after primary immunisation. Serum IgA titres against **(A)** WH-1 and **(B)** B.1.1.529 spike protein (n = 4−5 per group; each spot represents one sample). Bars represent the geometric mean ± geometric SD; *P < 0.05; **P < 0.01; ***P < 0.001; ns, P > 0.05.

Mucosal immunity is significantly associated with vaccine efficacy against COVID-19 at the early stages of infection. Mucosal immune response was evaluated by detecting spike protein-specific serum IgA titres with ELISA on day 35 after primary immunisation (**Figures 5A, B**). The results demonstrated that all five intranasal groups (in AdB > im ARO, in AdW > im ARO, in AdW > im ARW, in AdW, and in AdB) had high spike-specific IgA titres against the WH-1 strain on day 35 after primary immunisation, with GMTs of 1,939, 1,044, 648, 1,301 and 3,496, respectively (P>0.05; **Figure 5A**). The IgA GMTs of the eight intramuscular groups (im AdB > im ARO, im AdW > im ARO, im AdW > im ARW, im AdW, im AdB, im ARW > im ARW, im ARO > im ARO, and im ARW > im ARO) were 65, 46, 96, 14, 40, 194, 49 and 30, respectively. The IgA titres of vaccination groups were comparable with those of the in and im blank groups, indicating that no obvious mucosal immune response is induced by intramuscular vaccination.

Compared with spike-specific IgA titres against the WH-1 strain, higher spike-specific IgA titres were observed in all five intranasal groups (**Figure 5B**) against the B.1.1.529 strain, with IgA GMTs of 8,729, 1,128, 1,158, 1,991 and 7,576, respectively, showing a 3.5-, 0.1-, 0.8-, 0.5-, and 1.2-fold increase, respectively. The IgA GMTs of the eight intramuscular groups were 298, 170, 245, 151, 180, 230, 128 and 96, respectively. The IgA titres of vaccination groups were comparable with those of the in and im blank groups, suggesting that intramuscular vaccination may not trigger mucosal immune response.

### 3.3 Intranasal or intramuscular administration of AdB followed by ARO immunisation induces broad-spectrum neutralising activities against 2019-nCoV variants in mice

**Figure 6:**
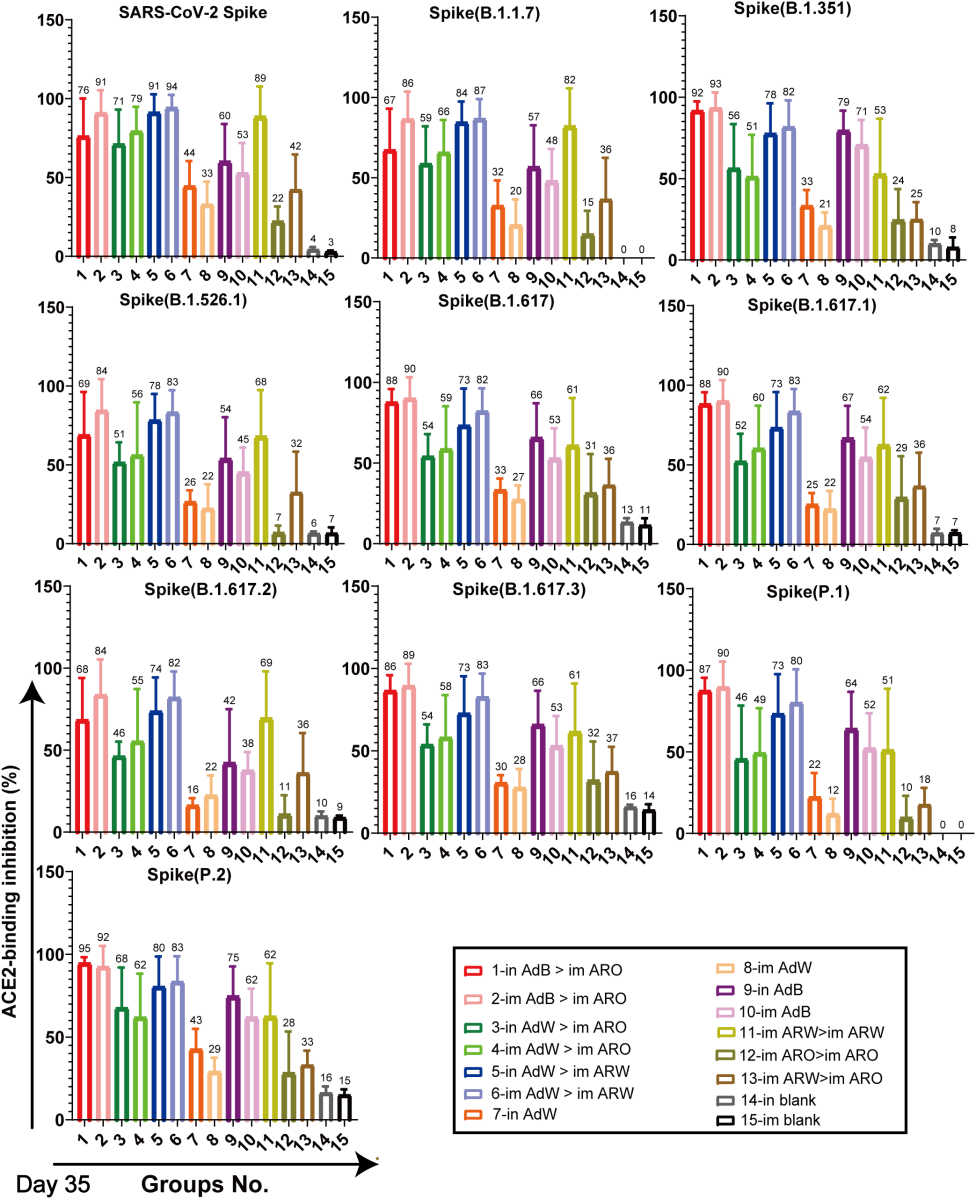
Neutralisation capacity of sera was observed by measuring the inhibition of binding between angiotensin-converting enzyme 2 (ACE2) and SARS-CoV-2 spike proteins on day 35 after primary immunisation. Spike proteins were from the SARS-CoV-2 prototype and B.1.1.7, B.1.351, B.1.526.1, B.1.617, B.1.617.1, B.1.617.2, B.1.617.3, P.1, and P.2 strains, respectively. Negative ACE2-binding inhibition rates are shown as zero (n = 5 per group). Bars represent the mean ± SD; numbers represent the mean of the corresponding group.

**Figure 7:**
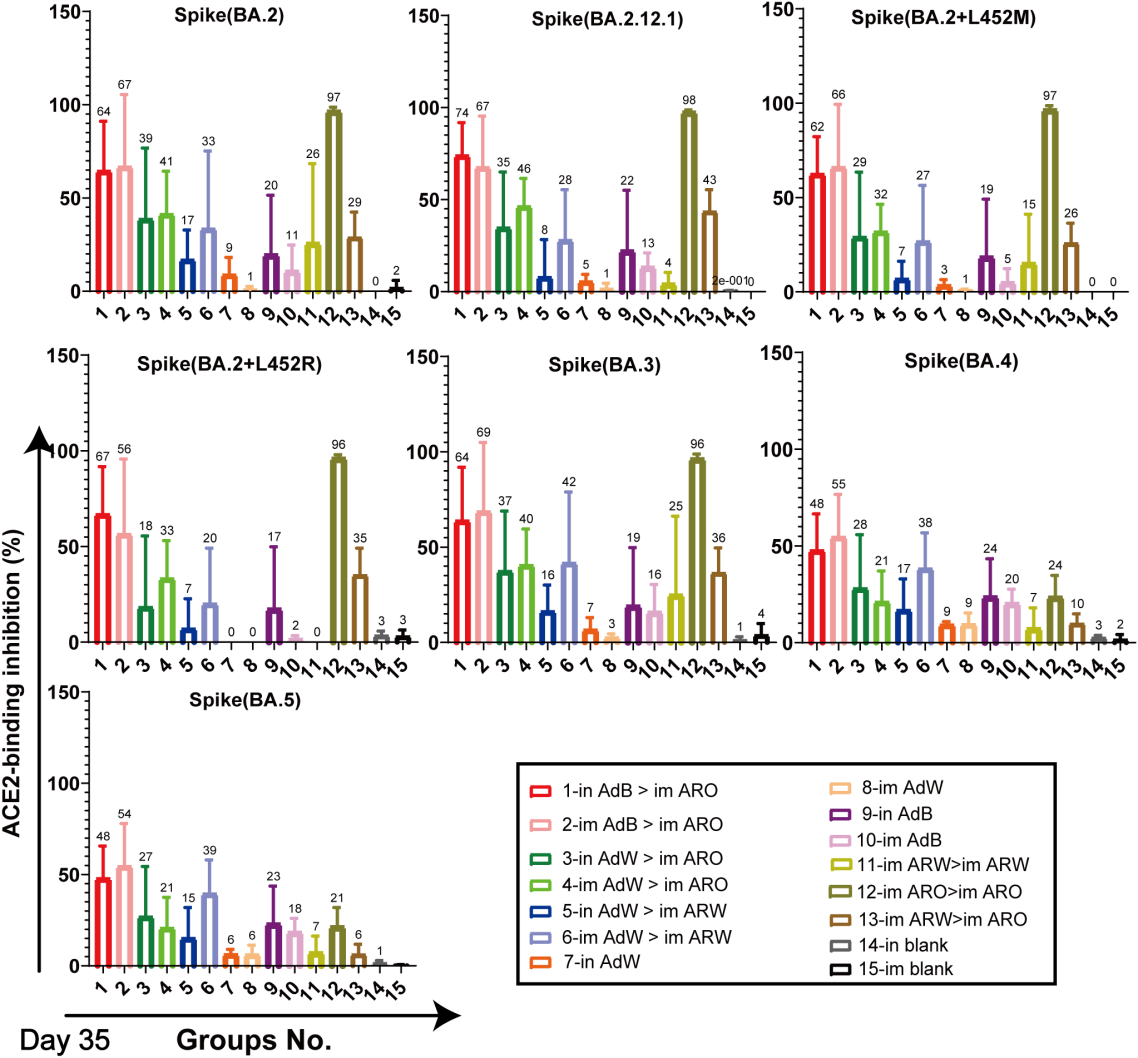
Neutralisation capacity of sera was observed by measuring the inhibition of binding between ACE2 and SARS-CoV-2 spike proteins on day 35 after primary immunisation. Spike proteins were from BA.2, BA.2.12.1, BA.2+L452M, BA.2+L452R, BA.3, BA.4, and BA.5 strains, respectively. Negative ACE2-binding inhibition rates are shown as zero (n = 5 per group). Bars represent the mean ± SD; numbers represent the mean of the corresponding group.

ACE2-binding inhibition (neutralising activity) rates (**Figures 6**, **7**) in serum against spike proteins from the 2019-nCoV prototype and variants (BA.5, BA.4, BA.3, BA.2+L452R, BA.2+L452M, BA.2.12.1, BA.2, P.2, P.1, B.1.617.3, B.1.617.2, B.1.617.1, B.1.617, B.1.526.1, B.1.351 and B.1.1.7) were detected using ACE2-binding inhibition (neutralisation) ELISA on day 35 after primary immunisation to assess the broad-spectrum neutralising activity. The ACE2-binding inhibition rates induced in all tested groups were consistent with the NAb titres determined using the VSV pseudovirus assay. In the heterologous prime-boost immunisation groups, in AdB > im ARO and im AdB > im ARO generated high and broad neutralisation activity against the spike protein of all the strains tested, with arithmetic mean values (AMVs) of ACE2-binding inhibition rates of 48–93%. The in AdW > im ARO and im AdW > im ARO groups produced relatively low neutralising activity against the spike protein of all tested strains, with ACE2-binding inhibition rate AMVs in the range of 18–74%. in AdW > im ARW and im AdW > im ARW produced high neutralising activities against the 2019-nCoV prototype and some variants (P.2, P.1, B.1.617.3, B.1.617.2, B.1.617.1, B.1.617, B.1.526.1, B.1.351 and B.1.1.7), with ACE2-binding inhibition rate AMVs in the range of 73–94%, but had low neutralising activity against Omicron lineages (BA.5, BA.4, BA.3, BA.2+L452R, BA.2+L452M, BA.2.12.1 and BA.2 variants), with ACE2-binding inhibition rate AMVs in the range of 7–42%. The im AdB > im ARO group possessed the highest and broadest neutralising activity, with inhibition rate AMVs of 54%, 55%, 69%, 56%, 66%, 67%, 67%, 92%, 88%, 88%, 81%, 89%, 89%, 82%, 93%, 85% and 90% against the BA.5, BA.4, BA.3, BA.2+L452R, BA.2+L452M, BA.2.12.1, BA.2, P.2, P.1, B.1.617.3, B.1.617.2, B.1.617.1, B.1.617, B.1.526.1, B.1.351 and B.1.1.7, respectively, which were as high and broad as those of the in AdB > im ARO group. The im and in AdB consistently induced higher ACE2-binding inhibition rates against the spike protein than in AdW and im AdW. Of all the strains tested, im ARW > im ARW induced higher ACE2-binding inhibition rates than im ARO > im ARO and im ARW > im ARO against 2019-nCoV prototype and some variants (B.1.1.7, B.1.351, B.1.526.1, B.1.617, B.1.617.1, B.1.617.2, B.1.617.3, P.1 and P.2), but lower rates against Omicron lineages (BA.5, BA.4, BA.3, BA.2+L452R, BA.2+L452M, BA.2.12.1 and BA.2 variants). The im ARO > im ARO group had low neutralising activities against the 2019-nCoV prototype and some variants (B.1.1.7, B.1.351, B.1.526.1, B.1.617, B.1.617.1, B.1.617.2, B.1.617.3, P.1 and P.2), with ACE2-binding inhibition rates equivalent to those of the in and im blank groups, but high rates against Omicron lineages (BA.5, BA.4, BA.3, BA.2+L452R, BA.2+L452M, BA.2.12.1 and BA.2 variants). The ACE2-binding inhibition rates on day 49 (**Figures S1, S2**) were consistent with those on day 35, which corresponded to NAb response levels on day 49 obtained using the VSV pseudovirus assay.

### 3.4 All AdW-, AdB-, ARW- and ARO-vaccinated groups exhibit strong cellular immunity against B.1.1.529, B.1.617.2 and WH-1 strains

**Figure 8:**
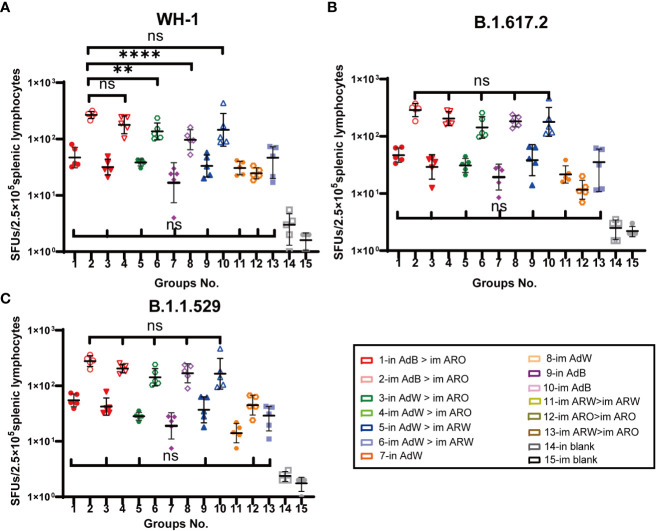
Cellular immune responses specific to SARS-CoV-2 spike proteins were measured 49 days after primary vaccination. Enzyme-linked immunospot (ELISpot) assays for IFN-γ after stimulation with the SARS-CoV-2 spike protein. Five mice per group were euthanised, and T-cell responses were measured. Lymphocytes were stimulated with **(A)** WH-1, **(B)** B.1.617.2, and **(C)** B.1.1.529 spike peptide pools spanning the entire spike protein sequence. Cells secreting IFN-γ were quantified using ELISpot assays (n = 5 per group; each point represents the mean number of spots from two wells per sample). Bars represent the geometric mean ± geometric SD; **P < 0.01; ****P < 0.0001; ns, P > 0.05.

Splenic lymphocytes were harvested on day 49 after primary immunisation and stimulated with the peptide pool that spans the spike proteins of the B.1.1.529, B.1.617.2 or WH-1 strain for 24 h, followed by IFN-γ ELISpot analysis (**Figures 8A−C**). The five AdB or AdW intramuscular-only vaccination groups (im AdB > im ARO, im AdW > im ARO, im AdW > im ARW, im AdW, and im AdB) had the highest T-cell responses against the WH-1 strain among all tested groups, with GMTs of spot forming units (SFUs) per 2.5 × 10^5^ splenic lymphocytes of 268, 178, 136, 97 and 145, respectively. The intranasal vaccination and homologous prime-boost groups (in AdB > im ARO, in AdW > im ARO, in AdW > im ARW, in AdW, in AdB, im ARW > im ARW, im ARO > im ARO, and im ARW > im ARO) had relatively low T-cell responses, with GMTs of SFUs per 2.5 × 10^5^ splenic lymphocytes of 47, 32, 38, 17, 33, 31, 25 and 39, respectively.

Similar results were observed against the B.1.617.2 (**Figure 8B**) and B.1.1.529 (**Figure 8C**) strains compared with the WH-1 strain. The GMTs of SFUs per 2.5 × 10^5^ splenic lymphocytes of the five AdB or AdW intramuscular-only vaccination groups (im AdB > im ARO, im AdW > im ARO, im AdW > im ARW, im AdW and im AdB) against the B.1.617.2 strain were 288, 204, 143, 183 and 179, respectively, showing no significant differences. The GMTs of SFUs per 2.5 × 10^5^ splenic lymphocytes of five AdB or AdW intramuscular-only vaccination groups (im AdB > im ARO, im AdW > im ARO, im AdW > im ARW, im AdW and im AdB) against the B.1.1.529 strain were 278, 205, 143, 168 and 165, respectively, showing no significant differences. The intranasal vaccination and homologous prime-boost groups had T-cell responses against the B.1.1.529, B.1.617.2 and WH-1 strains that were lower than those in the five AdB or AdW intramuscular-only vaccination groups.

### 3.5 Induction of skewed Th1 cell response in all AdW-, AdB-, ARW- and ARO-vaccinated groups

Next, we evaluated the Th1 skewing of T-cell responses specific to spike proteins. Splenic lymphocytes were harvested on day 49 after primary immunisation and stimulated with the peptide pool. Intracellular cytokine staining and MSD assays were performed to determine Th1-dominant T-cell responses against the different 2019-nCoV variants.

**Figure 9:**
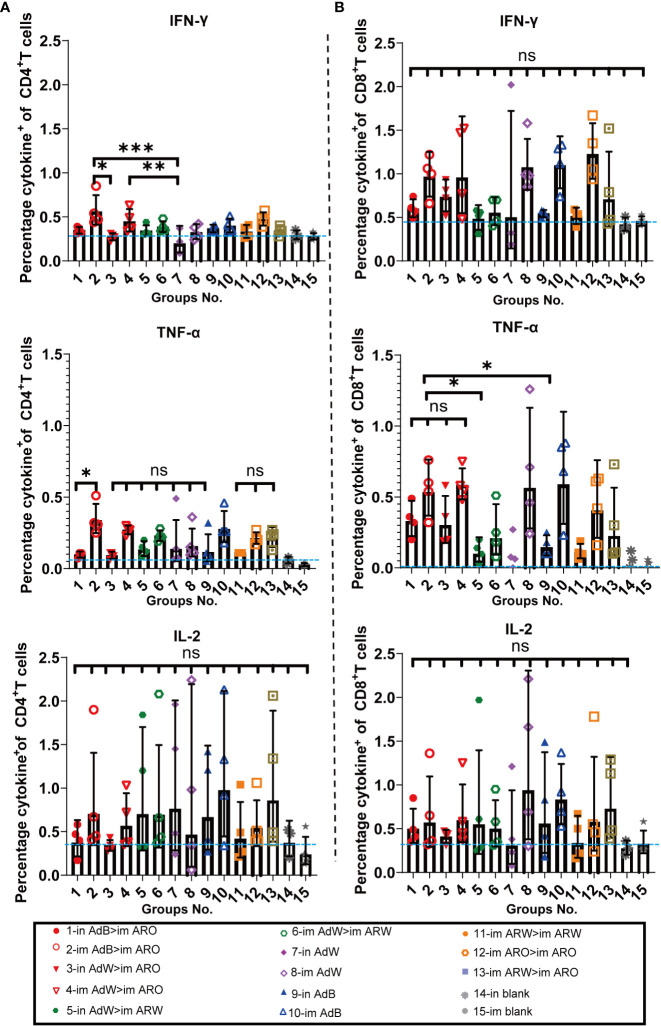
Th1/Th2 skewing was detected *via* intracellular cytokine staining on day 49 after primary immunisation. Percentage of spike protein-specific IFN-γ-, IL-2-, and TNF-α-positive memory **(A)** CD4^+^ T and **(B)** CD8^+^ T cells was measured on day 49 after primary immunisation (n = 4 per group; each point represents one sample). Bars represent the geometric mean ± geometric SD; *P < 0.05; **P < 0.01; ***P < 0.001; ns, P > 0.05. The blue dashed lines represent the blank value.

The intracellular cytokine staining results demonstrated that the ratios (%) of CD4^+^ (**Figure 9A**) and CD8^+^ T (**Figure 9B**) cells secreting Th1 typical cytokines (IL-2, TNF-α and IFN-γ) increased in all vaccination groups compared with the in and im blank groups, but the ratios (%) of CD4^+^ and CD8^+^ T cells secreting Th2 cytokines (IL-10 and IL-4) were not remarkably increased compared with the in and im blank groups. The proportions of IL-10 and IL-4 in CD8^+^ and CD4^+^ T cells are shown in **Figures S3A, B**. Overall, higher Th1 responses were found in the five AdB or AdW intramuscular-only vaccination groups compared with the other vaccination groups.

**Figure 10:**
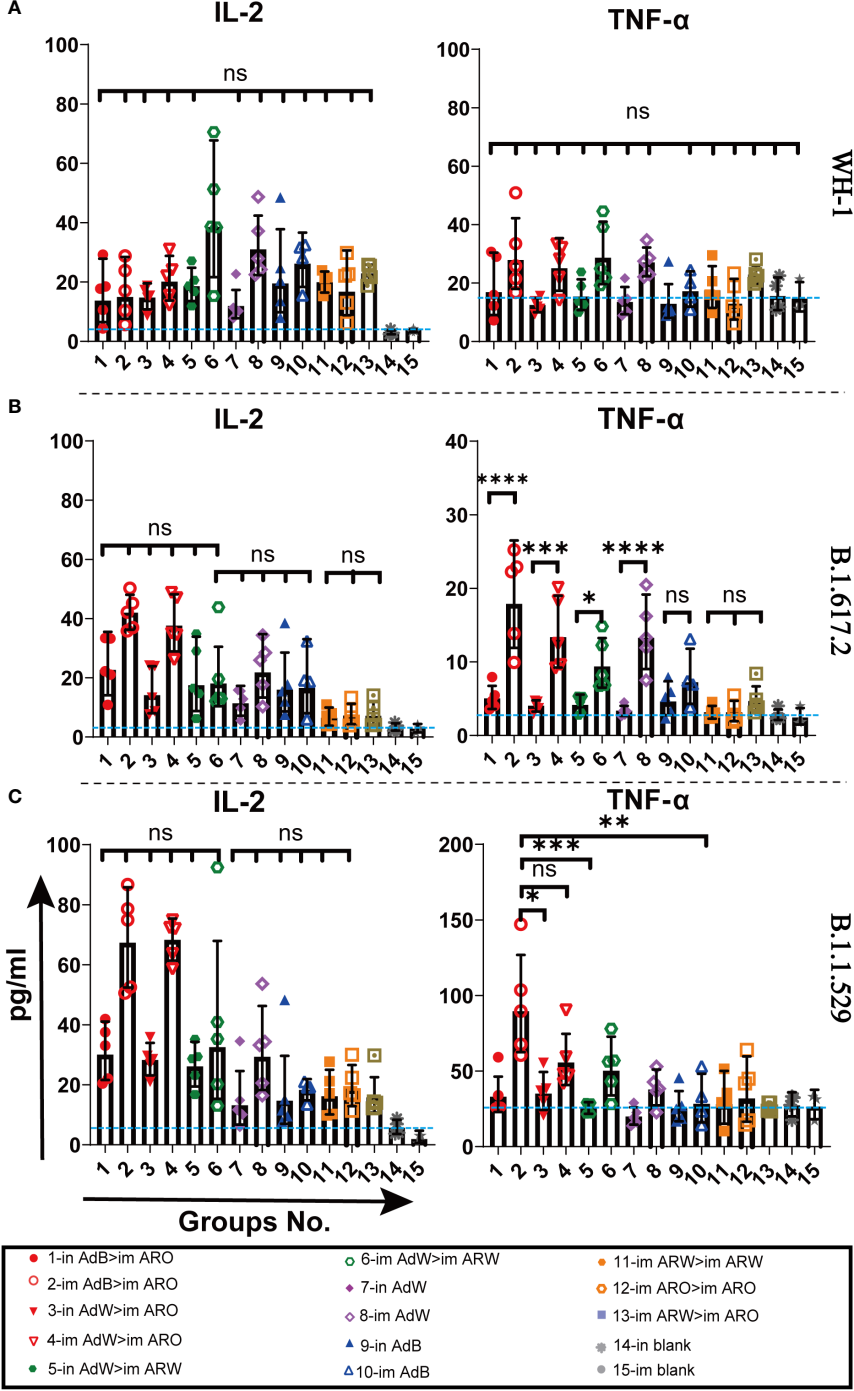
Th1/Th2 skewing in immunised mice was measured using Meso Scale Discovery (MSD) cytokine profiling. Lymphocytes were stimulated with **(A)** WH-1, **(B)** B.1.617.2, and **(C)** B.1.1.529 spike peptide pools spanning the entire spike protein sequence for 24 h. IL-2 and TNF-α levels in supernatants were measured (n = 5 per group; each point represents one sample). Bars represent the geometric mean ± geometric SD; *P < 0.05; **P < 0.01; ***P < 0.001; ****P < 0.0001; ns, P > 0.05. The blue dashed lines represent the blank value.

MSD cytokine profiling assays (**Figures 10A−C**) of TNF-α, IL-2, IL-4, and IL-10 were conducted to examine the functional preservation and polarisation of T cells specific to spike proteins against the B.1.1.529, B.1.617.2 and WH-1 strains. All vaccinated strategies elicited increased IL-2 and TNF-α concentrations, with higher levels in the five AdB or AdW intramuscular-only vaccination groups compared with the other groups, but lower concentrations of IL-10 and IL-4 that were comparable with the in and im blank groups (**Figures S4A−C**). These results suggest that the Th1-skewed, but not the Th2-skewed, response is considerably increased in all vaccination groups.

## 4 Discussion

The neutralisation and protective efficacy of different COVID-19 vaccines or monoclonal antibodies have markedly decreased with increasing VOCs (22–27). Different second-generation vaccines were developed to protect against multiple VOCs, but did not possess an ideal broad spectrum and cross-protection efficacy (28, 29). There is an urgent need to develop the optimal vaccination strategies for heterologous SARS-CoV2 variant-specific COVID-19 vaccines to improve their broad-spectrum protective efficacy against emerging 2019-nCoV variants. In this work, initial intramuscular vaccination with AdB followed by a booster vaccination with ARO exhibited the highest level of cross-reactive IgG, NAb responses, ACE2-binding inhibition rates, and Th1-based immune responses against the 2019-nCoV variants among all the strategies tested. However, this regimen did not induce local mucosal immune responses. Interestingly, intranasal administration of AdB followed by ARO not only induced systemic immune responses comparable to those induced by im AdB > im ARO, but also exhibited high levels of IgA and T-cell immune responses against multiple 2019-nCoV variants. Thus, in AdB > im ARO may be a promising immunisation strategy for enhancing the broad protection of chimpanzee adenovirus- and mRNA-based 2019-nCoV vaccines against VOCs.

Combining two different 2019-nCoV variant-specific vaccines was an effective strategy to increase the broad protection efficacy of chimpanzee adenovirus- and mRNA-based 2019-nCoV vaccines (11, 30, 31). Interestingly, we observed that heterologous prime-boost immunisation with AdB and ARO notably induced a broader and stronger systemic immune response than that induced by AdW and ARO or by AdW and ARW. Wang et al. (30) found that the heterologous Ad5-nCoV plus mRNA vaccine and homologous mRNA-Beta and mRNA-Omicron induced considerable cross-reactive neutralisation capacity against the prototype, Omicron, Delta and Beta variants in female C57BL/6 mice. Among these strategies, homologous prime-boost immunisation with mRNA-Beta and mRNA-Omicron induced the largest coverage of broad cross-neutralisation capacity, which was consistent with our results.

Numerous studies have indicated that 2019-nCoV Beta variant-specific COVID-19 vaccines induce broader spectrum immune responses against emerging 2019-nCoV variants than other variant-specific COVID-19 vaccines (9, 29). Our results demonstrated that single-dose immunisation with AdB could induce broader-spectrum NAb titres than AdW against different 2019-nCoV variants. Sun et al. (29) assessed the cross-reactive immune response capacity of recombinant COVID-19 protein vaccines that expressed spike protein RBD of the prototype and Alpha, Beta, Delta or Lambda variants in female C57BL/6 mice. The monovalent Beta-RBD vaccine generated higher and broader pseudovirus NAb responses against all five 2019-nCoV variants compared to the other tested vaccines. These findings are consistent with our previous results. The neutralisation site of most neutralising antibodies is in the RBD region of the spike protein (32). This observation may be attributed to the fact that B.1.351 strain (N501Y, E484K and K417T) has the same high-frequency mutations in RBD region as other 2019-nCoV variants such as N501Y of B.1.1.7, K417T, E484K and N501Y of P.1, E484A and N501Y of B.1.1.529, K417N, E484A and N501Y of BA.2, and K417N, E484A and N501Y of BA.4/BA.5, which are implicated in immune evasion and neutralising activity (2, 33, 34). The spike protein sequences containing these high-frequency mutation sites have been proposed as an effective strategy for designing universal COVID-19 vaccines (35).

Furthermore, we found that broader and more abundant cross-NAbs were generated by intranasal vaccination with AdB or AdW compared to intramuscular vaccination, probably due to the increased MIR induction with AdB or AdW *via* an intranasal route (18, 19, 36). Secretory IgA, as part of the MIR, plays an important role in preventing COVID-19 infection by limiting the virus at its point of entry in the upper respiratory tract (37–39). In addition, SIgA has non-specific neutralising properties (40), which may counteract the immune escape of emerging variants. However, we only evaluated IgA titres in the serum, which were inadequate to understand the role of SIgA after vaccination with AdB or AdW. Thus, the measurement of SIgA levels in bronchoalveolar lavage fluid is needed to characterize the MIR’s protective capacity more precisely after intranasal vaccination with AdB or AdW (36).

T cells plays crucial roles in secreting specific antiviral cytokines as well as recognising and killing infected cells (41). Moreover, T cells are involved in the humoral immune responses against 2019-nCoV in mice (42). Tarke et al. (43) found that the memory T-cell responses induced by numerous COVID-19 vaccines (Ad26.COV.S, BNT162b2, mRNA-1273 and NVX-CoV2373) were preserved and could cross-recognise early 2019-nCoV variants. However, the proportions of memory B cells and neutralising antibodies were markedly reduced in response to emerging variants. Our results also indicated that T-cell immune responses induced by AdW, AdB, ARW and ARO vaccines could cross-recognise 2019-nCoV VOCs. Meanwhile, all vaccination groups induced a Th1-biased cellular immune response, with higher concentrations of Th1-secreted TNF-α, IL-2 and IFN-γ in the five intramuscular-only vaccination groups than those in the intranasal vaccination and homologous prime-boost groups. These findings were consistent with our previous results (44).

Nevertheless, this study has few limitations. First, the neutralisation titres of other live 2019-nCoV variants were not measured due to the limited resources. NAb exhibits an immune protective effect on symptomatic COVID-19 infection (45), and additional experiments are warranted to verify the efficacy of the in AdB > im AdO route after challenge of other live 2019-nCoV variants. Second, we did not analyse SIgA levels in nasopharyngeal and bronchoalveolar lavage fluid, which could be useful in preventing 2019-nCoV variant infection. Third, the functional preservation of most T-cell responses could act as a second-level defence against diverse variants. However, we did not further assess whether the T-cell responses induced by AdW, AdB, ARW and ARO can cross-recognise other emerging 2019-nCoV variants.

In conclusion, primary immunisation with intranasal AdB followed by intramuscular ARO can induce broader spectrum, stronger local and systemic mucosal immune responses against different 2019-nCoV variants, indicating a promising strategy against VOCs and possibly emerging new variants in the future. Our findings provide a scientific basis for further development of broad-spectrum vaccines and immunisation strategies.

## Data availability statement

The original contributions presented in the study are included in the article/**Supplementary Material**. Further inquiries can be directed to the corresponding authors.

## Ethics statement

The animal study was reviewed and approved by the Institutional Animal Care and Use Committee of the National Institutes for Food and Drug Control is affiliated with the National Institute for Food and Drug Control.

## Author contributions

YL, JLi, and WH designed and supervised the study. XLi, JLiu, WL, ML, ZY, DZ, QP, YS, and LY performed the experiments. XLiu, JLiu, ZZ, ML, and QP analysed the data. YH, LS, HX, YW, GY, and XW provided administrative, technical, and material support. XLi, QP, JLiu and ML wrote the manuscript. WL and YL revised the manuscript. All authors contributed to the article and approved the submitted version.
