# Supplementary material for: Heterologous prime-boost immunisation with mRNA- and AdC68-based 2019-nCoV variant vaccines induces broad-spectrum immune responses in mice
Source: Front Immunol. 2023 Mar 15;14:1142394. doi: 10.3389/fimmu.2023.1142394 (PMC10050358; doi:10.3389/fimmu.2023.1142394)
Supplement: Supplementary file 1 [file DataSheet_1.docx]

Supplementary Material

Heterologous prime-boost immunisation with mRNA- and AdC68-based 2019-nCoV variant vaccines induces broad-spectrum immune responses in mice

Xingxing Li^1^†, Jingjing Liu^1,2^†, Wenjuan Li^1^†, Qinhua Peng^1^†, Miao Li^1^†, Zhifang Ying ^3^†, Zelun Zhang^1^, Xinyu Liu^1^, Xiaohong Wu^1^, Danhua Zhao^1^, Lihong Yang^1^, Shouchun Cao^1^, Yanqiu Huang^1^, Leitai Shi^1^, Hongshan Xu^1^, Yunpeng Wang^1^, Guangzhi Yue^1^, Yue Suo^1^, Jianhui Nie^4^, Weijin Huang^4*^, Jia Li^1*^, & Yuhua Li^1*^

*** Correspondence:**

Yuhua Li
[liyuhua@nifdc.org.cn](mailto:liyuhua@nifdc.org.cn)

Jia Li

[lijiarv@nifdc.org.cn](mailto:lijiarv@nifdc.org.cn)

Weijin Huang

[huangweijin@nifdc.org.cn](mailto:huangweijin@nifdc.org.cn)


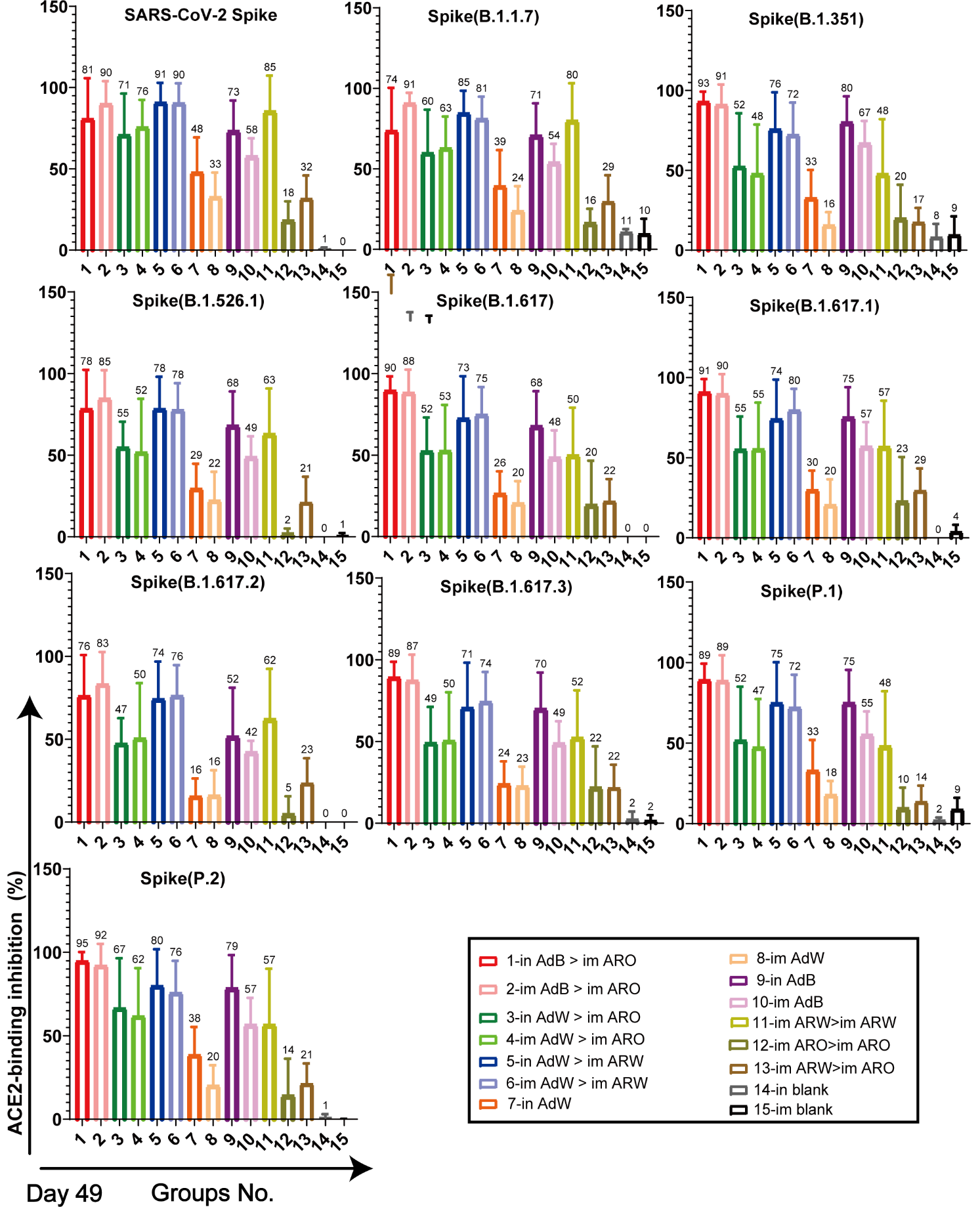


**Figure S1. Neutralisation capacity of sera was assessed by measuring the inhibition of binding between angiotensin-converting enzyme 2 (ACE2) and SARS-CoV-2 spike proteins on day 49 after primary immunisation.** Spike proteins were from the SARS-CoV-2 prototype and B.1.1.7, B.1.351, B.1.526.1, B.1.617, B.1.617.1, B.1.617.2, B.1.617.3, P.1, and P.2 strains, respectively. Negative ACE2-binding inhibition rates are shown as zero (n = 5 per group). Bars represent the mean ± SD; numbers represent the mean of the corresponding group.


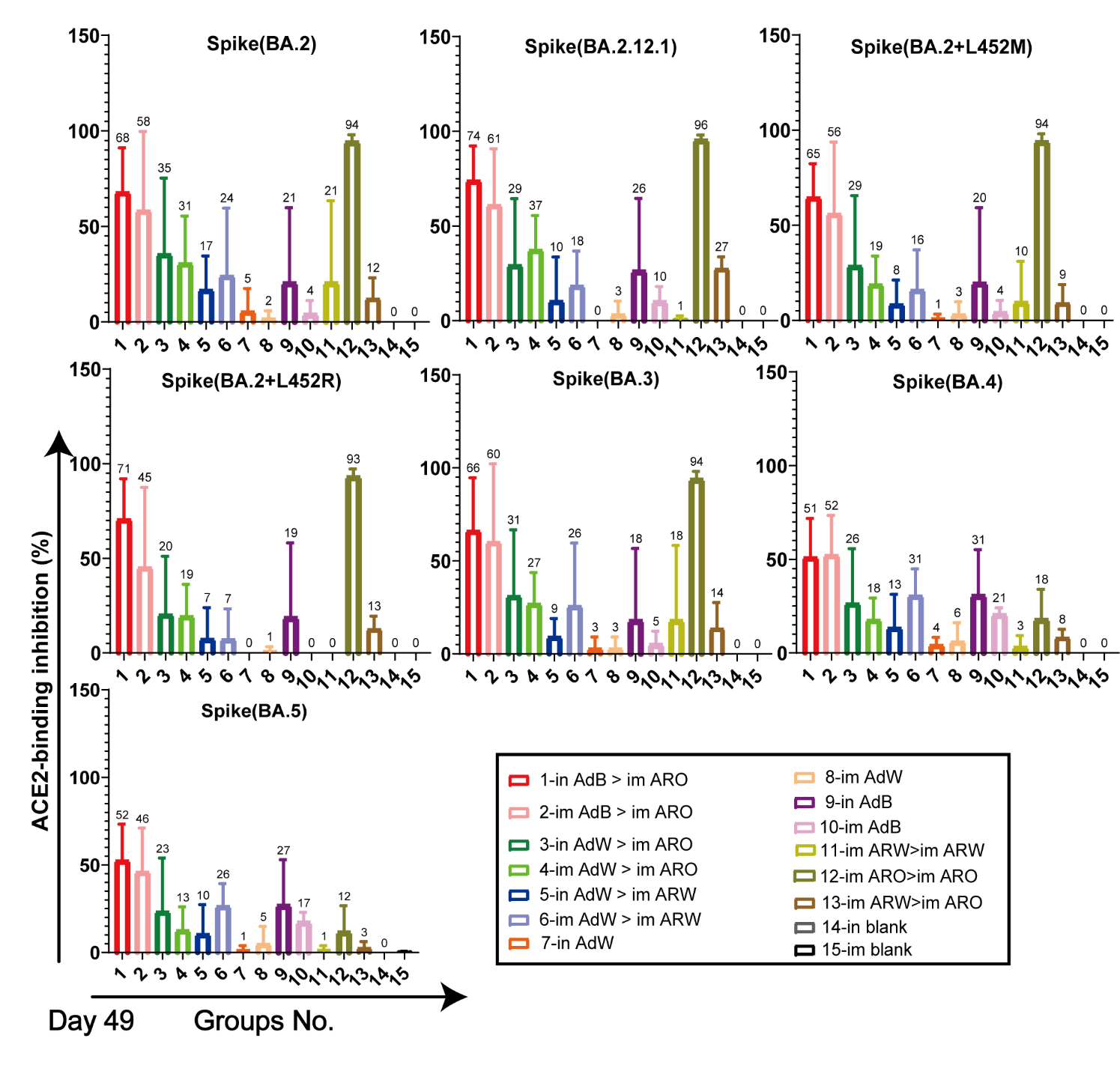


**Figure S2. Neutralisation capacity of sera was assessed by measuring the inhibition of binding between ACE2 and SARS-CoV-2 spike proteins on day 49 after primary immunisation.** Spike proteins were from BA.2, BA.2.12.1, BA.2+L452M, BA.2+L452R, BA.3, BA.4, and BA.5 strains, respectively. Negative ACE2-binding inhibition rates are shown as zero (n = 5 per group). Bars represent the mean ± SD; numbers represent the mean of the corresponding group.


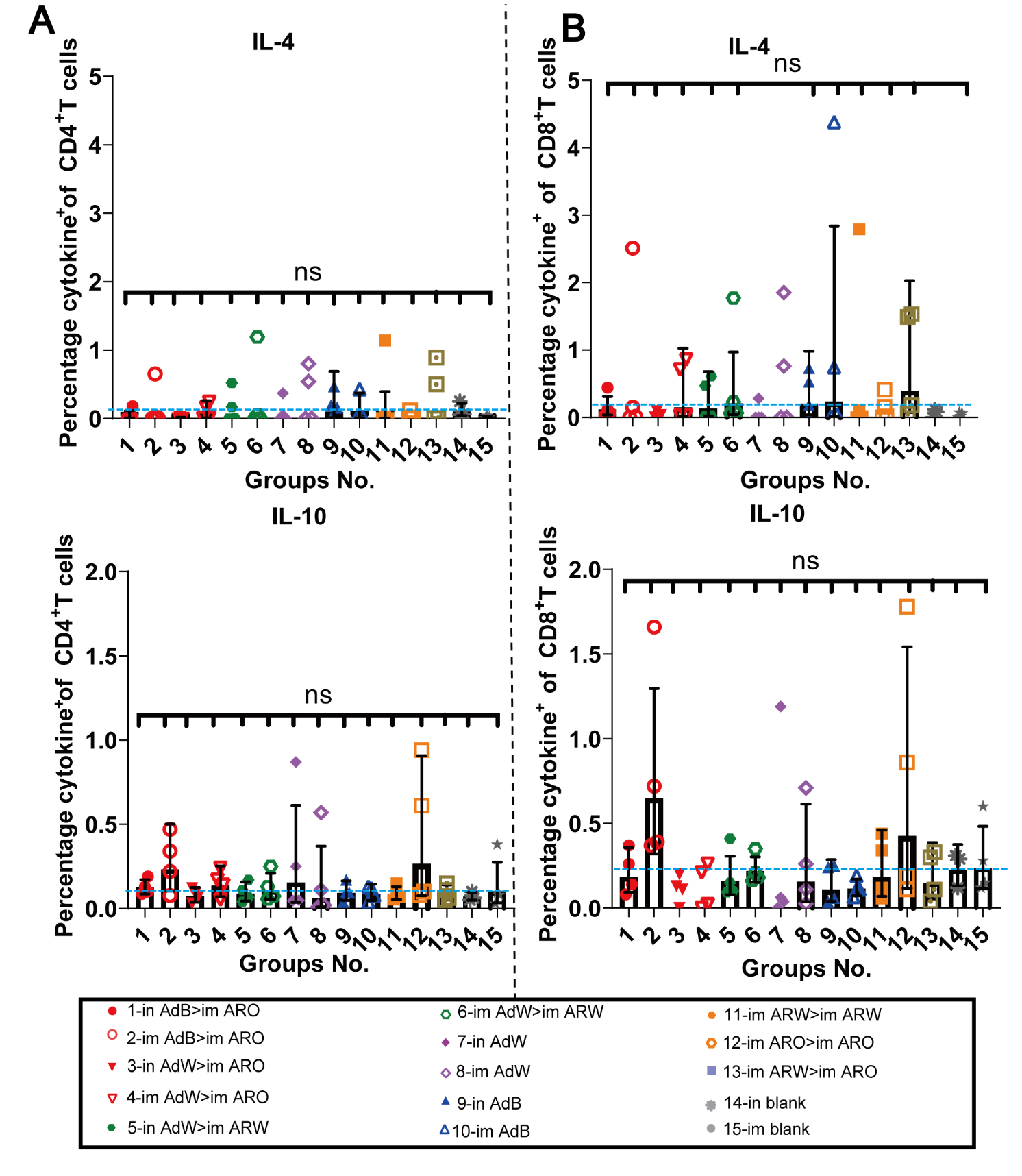


**Figure S3. Th1/Th2 skewing was detected by intracellular cytokine staining on day 49 after primary immunisation.** The percentage of spike protein-specific IL-4- and IL-10-positive (**A**) CD4^+^ T and (**B**) CD8^+^ T cells was measured on day 49 after primary immunisation (n = 4 per group; one spot represents one sample). Bars represent the geometric mean ± geometric SD; ns, P > 0.05. The blue dashed lines represent the blank value.


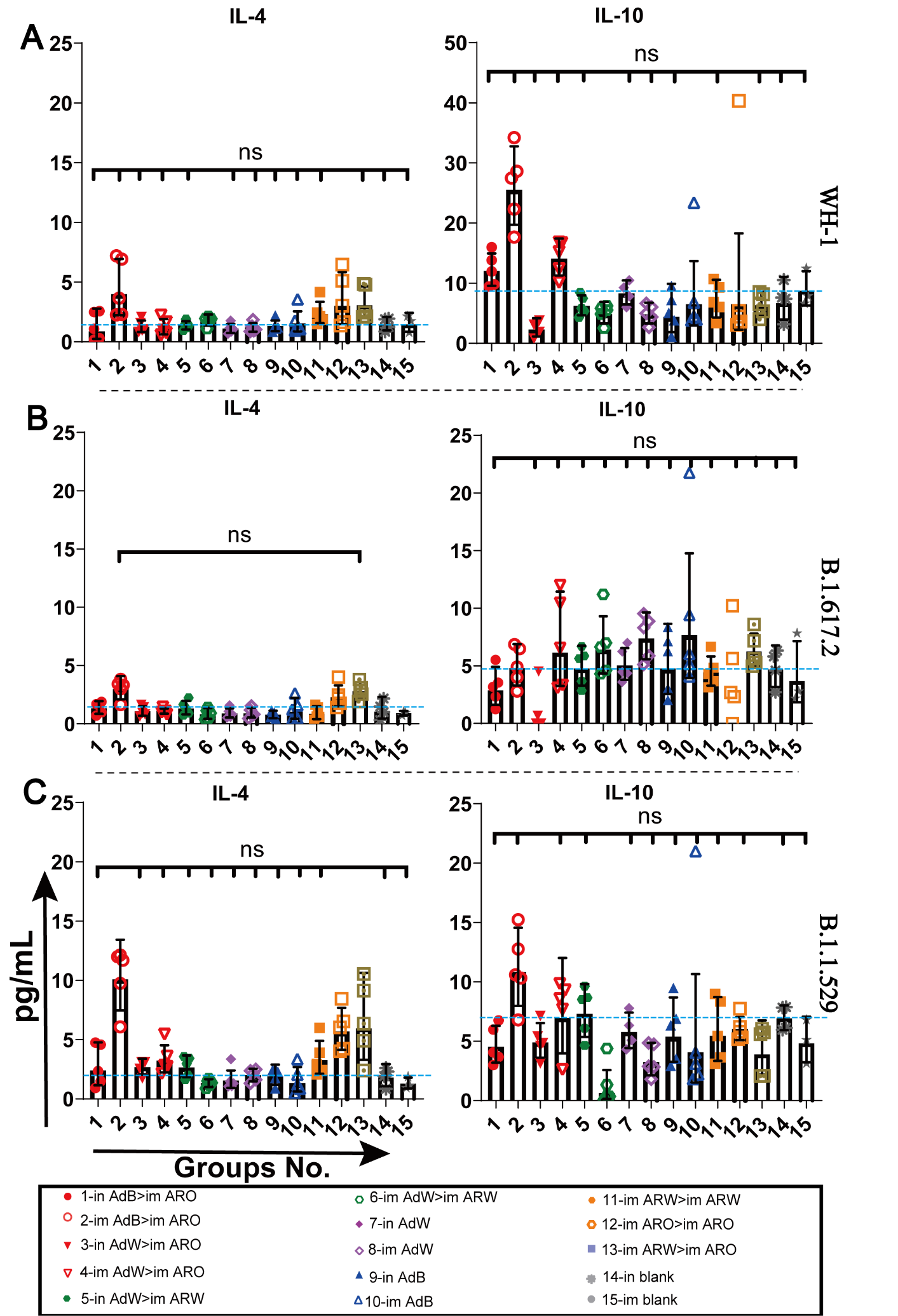


**Figure S4. Th1/Th2 skewing in immunised mice was measured using Meso Scale Discovery (MSD) cytokine profiling.** Lymphocytes were stimulated with (**A**) WH-1, (**B**) B.1.617.2, and (**C**) B.1.1.529 spike peptide pools spanning the entire spike protein sequence for 24 h. Levels of IL-4 and IL-10 in supernatants were measured (n = 5 per group; one spot represents one sample). Bars represent the geometric mean ± geometric SD; ns, P > 0.05. The blue dashed lines represent the blank value.
